# A Comparative Phylogenetic Analysis on the Chloroplast Genome in Different *Reynoutria japonica* Populations

**DOI:** 10.3390/genes13111979

**Published:** 2022-10-30

**Authors:** Jianhui Chen, Tiran Huang, Haili Fan, Fan Lin, Hongping Ma, Jie Cao, Tuanyao Chai, Lanqing Ma, Hong Wang

**Affiliations:** 1 College of Life Sciences, University of Chinese Academy of Sciences, Beijing 100049, China; 2 Beijing Advanced Innovation Center for Tree Breeding by Molecular Design, Beijing University of Agriculture, Beijing 102206, China; 3 Key Laboratory for Northern Urban Agriculture of Ministry of Agriculture and Rural Affairs, Beijing University of Agriculture, Beijing 102206, China

**Keywords:** *Reynoutria japonica*, chloroplast genome, genetic diversity, molecular marker, phylogeography, phylogenomics

## Abstract

*Reynoutria japonica* Houtt., a traditional medicine herb of the Polygonaceae family, has been used since ancient times in China due to its various pharmacological effects. Chloroplast genomes are conservative and play an essential role in population diversity analysis. However, there are few studies on the chloroplast genome of *R. japonica*. In this study, the complete chloroplast genomes of three *R. japonica* from different regions were performed by next-generation sequencing technology. The results revealed that the lengths of the three chloroplast genomes are between 163,371~163,372 bp, and they have a highly conserved structure with a pair of inverted repeat (IR) regions (31,121 bp), a large single-copy (LSC) region (87,571~87,572 bp), and a small single-copy (SSC) region (13,558 bp). In total, 132 genes were annotated, including 8 rRNA genes, 37 tRNA genes, and 87 protein-coding genes. The phylogenetic analysis strongly revealed that 13 populations of *R. japonica* form a monophyly, and *Fallopia multiflora* (Polygonaceae) is its closest species. The two species diverged at ~20.47 million years ago, and *R. japonica* in China could be further divided into two major groups based on genetic structure analysis. In addition, several potential loci with suitable polymorphism were identified as molecular markers. Our study provides important genetic resources for further development and utilization of *R. japonica* germplasm, as well as some new insights into the evolutionary characteristics of this medicinal plant.

## 1. Introduction

*R. japonica* Houtt., also known as *Polygonum cuspidatum* Sieb. et Zucc., *Fallopia japonica* (Japanese knotweed), and *HuZhang*, is a perennial herb in the Polygonaceae family and has been used as a traditional Chinese medicine for thousands of years [1]. *R. japonica* is rich in stilbenes, flavonoids, and anthraquinones [2], which play important roles in human health, functioning in anti-inflammation, anti-infection, and anticancer [1,3,4], and has been listed in the Pharmacopoeia of the People’s Republic of China since 1977. At present, it is in the recommendation of “Diagnosis and Treatment Protocol for COVID-19 (Trial Version 7).” As a medicinal plant with fast growth, easy adaptation, and high tolerance [5,6], *R. japonica* is considered an invasive species in Europe, causing enormous economic losses [7]. Meanwhile, this medicinal/invasive plant also has the ability to accumulate high levels of heavy metals [8]. The allelopathy effect of *R. japonica* may help it to rapidly establish an environment conducive to its own growth in new habitats and to interrupt the regeneration process in native plant species [9]. Moreover, the places with *R. japonica* usually form monospecific stands [10]. Hence, it is desirable to conduct a molecular-level analysis of the genetic diversity and variation range of *R. japonica* populations.

Chloroplast is a critical site for photosynthesis and has its own DNA and genetic system [11]. The chloroplast genomes are ubiquitous in plants and possess a typical quadripartite structure [12], including a large single-copy (LSC) and a small single-copy (SSC), as well as a pair of inverted repeats (IRs), more conserved than nuclear and mitochondrial genomes [13]. In addition, chloroplast genomes are small and, with a length of 120 kb to 160 kb [14], are easy to assemble and are of diverse value such as for elucidating phylogenetic relationships, resolving seemingly intractable problems in traditional taxonomy [15], clarifying interspecific and intraspecific variation [16], and identifying species [17]. With the advances in next-generation sequencing technology, more and more species of chloroplast genomes in the Polygonaceae family have been obtained, including *Fagopyrum esculentum*, *Fagopyrum tataricum*, *Fagopyrum dibotrys* [13,18,19,20], *F. multiflora* [21], and other species sequences published in public databases. In *Flora of China*, *R. japonica* was separated from *Polygonum* and considered to belong to a new genus, *Reynoutria* Houtt. Several studies using chloroplast genomes revealed that *R. japonica* and *F. multiflora* had a close genetic relationship [22,23]. However, the intraspecific and population structure among *R. japonica* has not yet been clarified till now. As a result, it is of great interest to provide its genomic data in terms of intraspecific and population structure of *R. japonica*.

Currently, most research into this herbal medicine has focused on pharmacological activities [24], biosynthesis, and regulation of active compounds [25,26,27]. Few studies are available to systematically compare the characteristics and phylogenetic relationships of *R. japonica* across different regions of China based on chloroplast genomes [23]. In this study, chloroplast genomes of *R. japonica* from three different Chinese regions were sequenced by us, and a total of 13 chloroplast genomes of *R. japonica* were obtained, together with other chloroplast genomes published in public databases. Then, a series of bioinformatics analyses was carried out, including identifying molecular markers that could be used to discriminate between different germplasm populations of *R. japonica* and exploring the evolutionary time and geographical distribution characteristics of *R. japonica* in China. The chloroplast genome resources assembled in this study could also serve as some material foundation for the further development and utilization of *R. japonica* germplasm.

## 2. Materials and Methods

### 2.1. Materials and DNA Extraction

The seeds of *R. japonica* were collected from Lichuan City (*Rj. LiChuan*), Hubei Province of China; Shandong Province of China (*Rj. ShanDong*); and Beijing (*Rj. BeiJing*), China. All sample seedings were propagated in plastic pots containing a soil/vermiculite mixture (1:1) and the growth environments, as described in a previous study [25]. The fresh leaves of *R. japonica* were quickly frozen with liquid nitrogen immediately after picking and cleaning. Total whole genomic DNA was extracted with Super Plant Genomic DNA Kit (polysaccharide- and polyphenolics-rich) (Tiangen, Beijing, China), and purity and integrity of the extracted total genomic DNA were detected by 1% agarose gel electrophoresis, and the total concentration was estimated by Qubit DNA Assay Kit in Qubit 3.0 Fluorometer (Invitrogen, Waltham, MA, USA). The qualified samples were selected for subsequent experiments.

### 2.2. Chloroplast Genome Assembly and Annotation

The raw sequencing reads were obtained by the company (Novogene, Beijing, China). A total amount of 0.2 μg DNA per sample was used as input material for the DNA library preparations. Sequencing library was generated using NEB Next Ultra DNA Library Prep Kit for Illumina (San Diego, CA, USA) following manufacturer’s recommendations, and index codes were added to each sample. The clustering of the index-coded samples was performed on a cBot Cluster Generation System using Illumina PE Cluster Kit (Illumina, San Diego, CA, USA) according to the manufacturer’s instructions. After cluster generation, the DNA libraries were sequenced on Illumina NovaSeq 6000 platform (Illumina, San Diego, CA, USA), and 150 bp paired-end reads were generated. The raw data contained adapter contamination, low-quality nucleotides, and unrecognizable nucleotide (N). We used fastp v0.19.7 [28] to obtain high-quality reads. The specific process was as follows: (1) Discard a pair of reads if either one read contains adapter contamination. (2) Discard a pair of reads if more than 10% of bases are uncertain in either one read. (3) Discard a pair of reads if the proportion of low-quality (Phred quality < 5) bases is over 50% in either one read. Then, the high-quality reads were used to assemble the whole chloroplast genome with GetOrganelle [29]. WTDBG [30] was used to polish the chloroplast genome with clean reads. BWA v0.7.17-r1188 [31] was used to map the clean data to assemble chloroplast genome, and samtools v1.9 [32] was used to calculate the sequencing depth of each locus. The complete chloroplast genome sequences of three *R. japonica* were annotated via GeSeq [33]. All three annotation results were manually curated. Finally, the resulting plastid genome maps were drawn with OGDRAW [34].

### 2.3. Chloroplast Genomes Feature Analysis

We compared the boundaries of the LSC, IR, and SSC regions of *R. japonica* chloroplast genomes by IRscope [35]. All the shared genes of *R. japonica* were extracted and aligned with MAFFT v7.503 [36], including 75 protein-coding genes, 4 rRNA, and 28 tRNA. Then, the alignments of each gene were used to evaluate nucleotide diversity (Pi) using DnaSP v6.0 [37]. The mVISTA program [38] in Shuffle-LAGAN mode [39] was used to analyze the intraspecific variation with *Rj. BeiJing* acting as the reference genome. For the protein-coding genes codon usage, the relative synonymous codon usage (RSCU), codon adaption index (CAI), codon bias index (CBI), frequency of optimal codons (FOP), GC contents of silent third codon position (GC3s), and effective codon number (ENC) were determined by CodonW v1.4.4 (available online: https://codonw.sourceforge.net/, accessed on 1 September 2022).

### 2.4. Chloroplast Phylogenetic and Divergence Time Estimation

In addition to the chloroplast genomes of *R. japonica* from three Chinese regions obtained by us, five samples of *R. japonica* were provided by [23], and another five chloroplast genomes data were obtained from the NCBI (Accession: NC_059800.1, MW411186.1, MT955361.1, MK381448.1, and MT301955.1). *F. multiflora* (MK330002.1), *Fagopyrum dibotrys* (MF491390.1), *Fagopyrum tataricum* (KM201427.1), Fagopyrum esculentum (EU254477.1), *Fallopia convolvulus* (OK0409957.1), *Polygonum aviculare* (NC_058892.1), and *Vitis vinifera* (NC_007957.1) were used as an outgroup to root the phylogenetic tree.

Phylogenetic analysis based on the shared protein-coding genes (PCGs) and MAFFT v7.503 [36] was used to align the shared PCGs under default parameters. A total of 74 PCGs were used in the concatenated dataset, which were: *atpA*, *atpB*, *atpE*, *atpF*, *atpH*, *atpI*, *ccsA*, *cemA*, *clpP*, *infA*, *ndhA*, *ndhB*, *ndhC*, *ndhD*, *ndhE*, *ndhF*, *ndhG*, *ndhH*, *ndhI*, *ndhJ*, *ndhK*, *petA*, *petB*, *petD*, *petG*, *petL*, *petN*, *psaA*, *psaB*, *psaC*, *psaI*, *psbA*, *psbB*, *psbC*, *psbD*, *psbE*, *psbF*, *psbH*, *psbI*, *psbJ*, *psbK*, *psbM*, *psbN*, *psbT*, *psbZ*, *rbcL*, *rpl14*, *rpl16*, *rpl2*, *rpl20*, *rpl22*, *rpl32*, *rpl33*, *rpl36*, *rpoA*, *rpoB*, *rpoC1*, *rpoC2*, *rps11*, *rps12*, *rps14*, *rps15*, *rps16*, *rps18*, *rps19*, *rps2*, *rps3*, *rps4*, *rps7*, *rps8*, *ycf1*, *ycf2*, *ycf3*, *ycf4*. Nucleotide substitution model selection was estimated with ModelTest-NG [40]. The GTR + G4 model was chosen as the best-fit model based on Bayesian information criterion. Maximum likelihood (ML) phylogenetic inference tree was performed by RAxML-NG v0.9.0 [41] with 1000 bootstrap replicates. The Bayesian inference (BI) tree was generated in MrBayes v3.2.7a [42], running for 2,000,000 generations, and the parameters were sampled every 1000 generations. The best-fit model (GTR + I+G) was selected by Akaike information criterion in MrModeltest v2.4 (available online: https://github.com/nylander/MrModeltest2, accessed on 1 September 2022).

Species divergence times were estimated by mcmctree program in PAML v4.8a [43] based on the maximum likelihood phylogenetic inference tree, the 74 PCGs, and calibrated time from TimeTree [44]. Specifically, pairwise divergence time for *R. japonica* and *F. multiflora* was 14.6~43.9 million years ago (MYA), 25.5~55.2 MYA for *R. japonica* and *F. tataricum*, and 112.4~125.0 MYA for *R. japonica* and *V. vinifera*. The results were treated with FigTree v1.4.4.

### 2.5. Genetic Diversity and Population Differentiation of R. japonica

The whole chloroplast genomes of the 13 *R. japonica* were aligned by MAFFT v7.503 [36]. The 13 whole chloroplast genomes had an aligned length of 163,725 bp. A total of 245 mutation sites were screened in MEGA v7.0 [45], including 142 singleton and 103 parsimony informative sites, which were used to analyze population structure with an admixture model-based clustering method implemented in STRUCTURE v2.3.4 [46]. We conducted 20 independent runs for each value from K = 1~10 to estimate the “true” number of clusters in 200,000 Markov chain Monte Carlo cycles following a burn-in step of 500,000 iterations. The most likely number of clusters was determined via the online website STRUCTURE HARVESTER [47]. Then, CLUMPP v1.1.2 and the greedy algorithm were used to align multiple runs of STRUCTURE for the best value of K [48]. In addition, the 245 mutation sites were also used to perform principal component analysis (PCA) to assess genetic structure.

## 3. Results

### 3.1. Chloroplast Genome Assembly and Annotation of R. japonica

**Figure 1:**
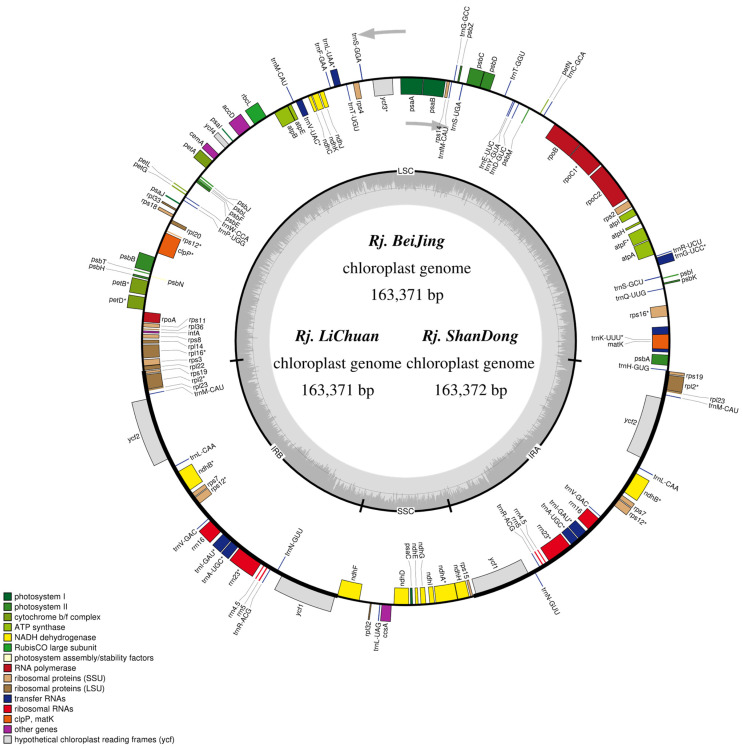
Gene map of the three *R. japonica* chloroplast genomes. Genes drawn inside the circle are transcribed clockwise, and genes outside are transcribed counter-clockwise. Different colors encode genes belonging to different functional groups. The area in darker gray and lighter gray in the inner circle corresponds to GC content and AT content, respectively.

The whole chloroplast genome of the three sequenced *R. japonica* comprised a typical covalently closed and double-stranded circular molecule (Figure 1). The results indicated that the complete chloroplast genome had a length of 163,371 bp (*Rj. BeiJing*), 163,371 bp (*Rj. LiChuan*), and 163,372 bp (*Rj. ShanDong*), respectively. The structure of the chloroplast genome included a large single-copy (LSC) region, a small single-copy (SSC) region, and two inverted repeat (IR) regions. The coverage of sequencing depth ranged from 2677.20× to 3740.61× (Table S1). The length of the LSC region was 87,571 bp (*Rj. BeiJing* and *Rj. LiChuan*) and 87,571 bp (*Rj. ShanDong*), respectively. The length of the SSC and IR regions in three *R. japonica* were 13,558 bp and 31,121 bp, respectively (Table 1). The total GC content of the three plastomes ranged from 37.52% to 37.53%, and the three plastomes encoded 79 protein-coding genes, 30 tRNA genes, and 4 rRNA genes (Table 1). In addition, 19 genes contained introns, 17 genes had a single intron, and only *ycf3* and *clpP* genes had 2 introns. The intron in *trnK*-UUU contained *matK* (Figure 1). Two genes, *psbL* and *ndhD*, were transcribed with ACG because of RNA editing. The obtained whole chloroplast genome sequences of the three *R. japonica* were submitted to the NCBI database with the GenBank accession numbers OP583946 (*Rj. BeiJing*), OP583947 (*Rj. LiChuan*), and OP583948 (*Rj. ShanDong*).

### 3.2. Features of the R. japonica Chloroplast Genomes

**Figure 2:**
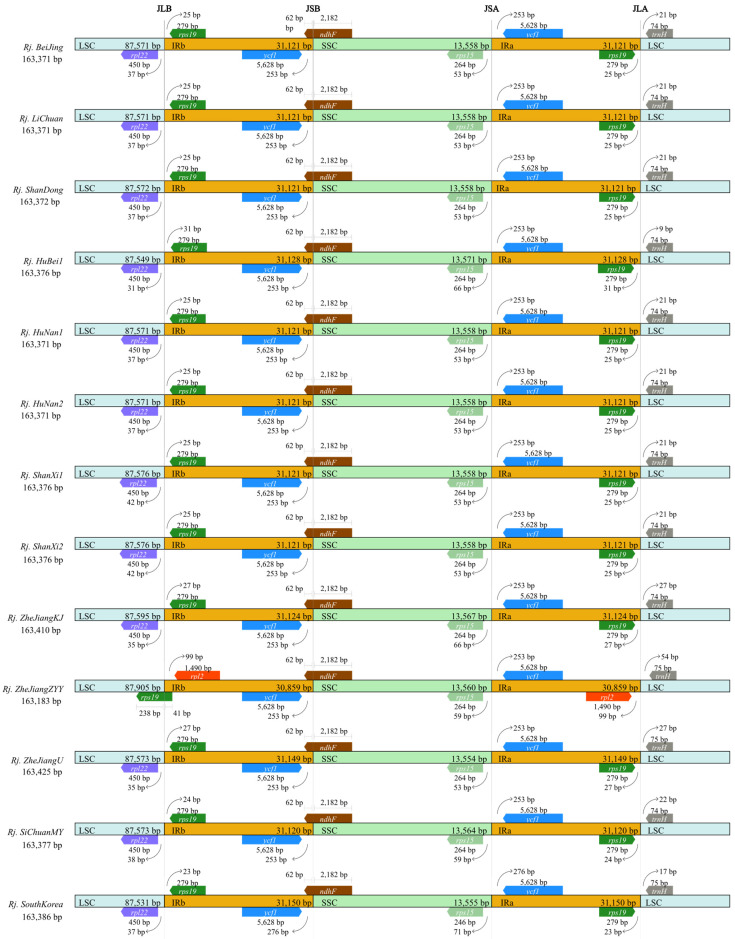
Comparison of the borders of large single-copy (LSC), small single-copy (SSC), and inverted repeat (IR) regions among the 13 *R. japonica* chloroplast genomes. Genes are denoted by colored boxes, and the gaps between the genes and boundaries are proportional to the distances in bps.

The chloroplast genome length of 13 *R. japonica* ranged from 163,183 (*Rj. ZheJiangZYY*) to 163,425 bp (*Rj. ZheJiangU*) (Table S2), with a GC content of 37.52~37.54%. The LSC region varied the most in length (87594.92 ± 90.65 bp, mean ± standard deviation), but the GC content varied little (35.63 ± 0.0060%). The SSC region was the second most variable in length at 31105.92 ± 71.99 bp, and the difference in GC content variation was at 41.22 ± 0.016%. Compared with the LSC and IRs regions, the SSC region showed the smallest range of length variation (13559.77 ± 4.59 bp), while the GC content showed the greatest variation (32.76 ± 0.26%) (Table S2). The border positions of JLB (junction of LSC and IRb), JSB (junction of SSC and IRb), JSA (junction of SSC and IRa), and JLA (junction of LSC and IRa) among the 13 *R. japonica* samples were compared. The above results revealed the length of IR regions with some contraction or expansion ranging from 30,859 to 31,150 bp (Figure 2). Some notable differences were found in the JLB junction point; for example, the JLBs of the 12 *R. japonica* populations were located between *rpl22* and *rps19*, except for *Rj. ZheJiangZYY*, whose JLB was located in *rps19*, and the length of *rps19* in IRb was 41 bp. There was only one copy of the *rps19* gene in *Rj. ZheJiangZYY*, while there were two copies in the other samples. It is consistent in *R. japonica* that the *ndhF* gene stretched over JSB, with 62 bp in the IRb region and 2182 bp in the SSC region. The JSA junction point was located between *rps15* and *ycf1*; the JLA junction point was located between *rps19* and *trnH* (Figure 2). The mVISTA results revealed that the whole chloroplast genomes of the 13 *R. japonica* samples were relatively similar (Figure S1), indicating that the chloroplast genome in *R. japonica* is highly conserved. The mVISTA results also indicated that the variations in these 13 samples could be divided into two groups, one with less variation than *Rj. BeiJing,* and the other with the opposite pattern (Figure S1).

### 3.3. Molecular Marker Identification

**Figure 3:**
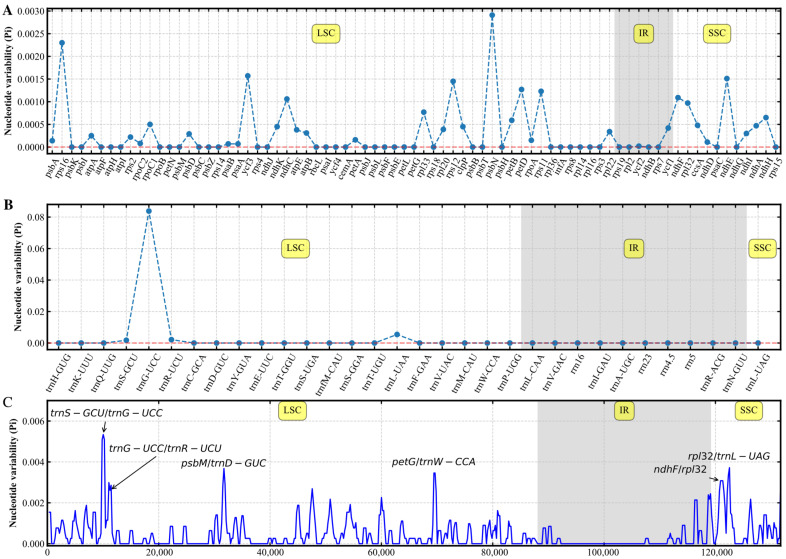
Gene nucleotide diversity (Pi) values of the 13 *R. japonica.* The 75 protein-coding genes (**A**) and 32 nonprotein-coding genes (**B**). *X*-axis, gene name; *Y*-axis, Pi value. (**C**) Six regions with the highest Pi values were masked based on alignment. Window length, 600 bp; step size, 200 bp. LSC, large single copy; IR, inverted repeats; SSC, small single copy.

To investigate the genetic diversity of various *R. japonica* and exploit potential polymorphic genes for identifying the specificity of *R. japonica* in different regions, gene nucleotide diversity (Pi) values of the 13 samples in *R. japonica* were calculated (Figure 3). In the protein-coding and nonprotein-coding genes, the Pi value ranged from 0 to 0.08381 (Figure 3A,B). In addition, the sliding window method was used to calculate the Pi values in each chloroplast genomes region using a window size of 600 bp (Figure 3C). The results indicated that most variations in the chloroplast genomes occurred in the LSC region. Gene nucleotide diversity was lowest in the IR region. Therefore, the IR region was more conserved than the other two regions. Six peaks were identified in the 13 *R. japonica*, with Pi values > 0.0025 (Figure 3C). All peaks were located in intergenic regions. Among them, *trnS-GCU*/*trnG-UCC*, *trnG-UCC*/*trnR-UCU*, *psbM*/*trnD-GUC,* and *petG*/*trnW-CCA* were located in the LSC region, and *ndhF*/*rpl32* and *rpl32*/*trnL-UAG* were located in the SSC region (Figure 3C). The results showed that the 13 *R. japonica* samples manifested, in general, a lower degree of nucleotide diversity.

### 3.4. Indices of Codon Usage

**Figure 4:**
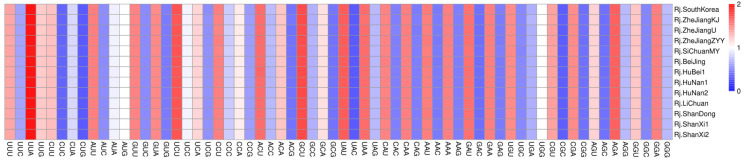
The RSCU values of all the concatenated protein-coding genes for the 13 *R. japonica* chloroplast genomes.

In order to analyze the codon usage of *R. japonica* chloroplast genomes, 75 shared protein-coding genes were extracted and connected as a concatenated dataset for each chloroplast genome. These sequences ranged from 64,275 bp to 64,665 bp, encoding 21,425~21,555 codons. The Leu was encoded by the highest number of codons (2275~2285), while the Cys was by the lowest (228~235). The relative synonymous codon usage (RSCU) values of all the codons ranged from 0.36 to 1.92 (Figure 4, Table S3). The four most frequently used codons with a usage count of more than 800 were AUU (Ile), GAA (Glu), AAA (Lys), and UUU (Phe). The results indicated that the CAI, CBI, FOP, GC3s, and ENC values were similar among the 13 *R. japonica* samples (Table S4).

### 3.5. Plastid Phylogenomics of R. japonica

**Figure 5:**
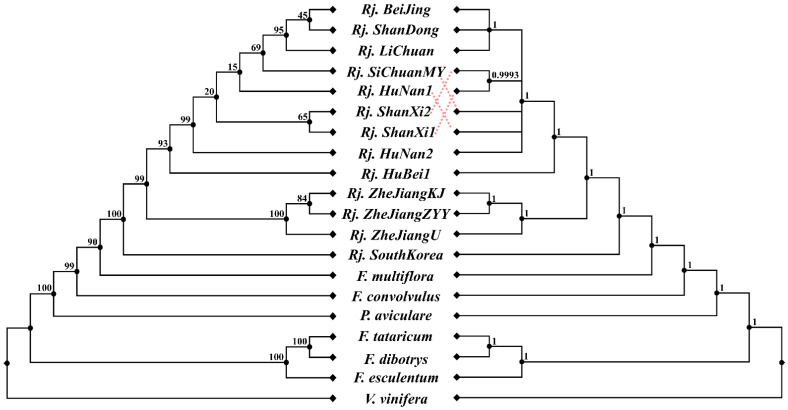
Phylogenetic trees based on protein-coding genes. Maximum likelihood (ML) phylogenetic inference tree (**left**) and Bayesian inference (BI) tree (**right**); ML bootstrap support values/Bayesian posterior probabilities are shown at each node; *F. convolvulus, Fallopia convolvulus; F. tataricum, Fagopyrum tataricum; F. dibotrys, Fagopyrum dibotrys; F. esculentum, Fagopyrum esculentum*.

**Figure 6:**
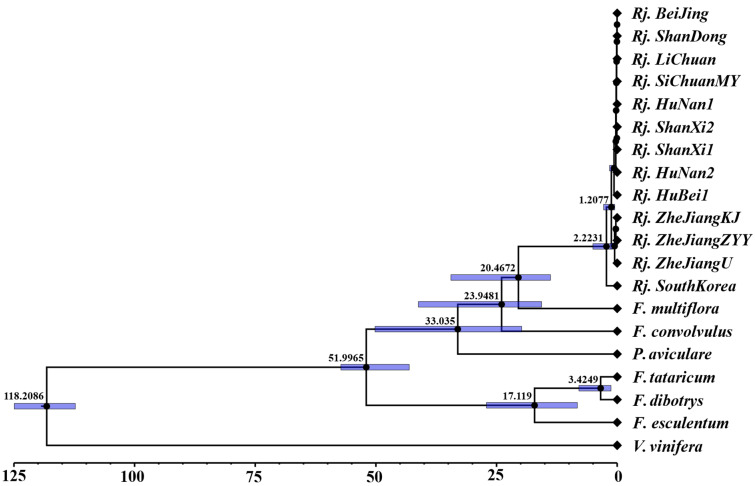
Divergence times of *R. japonica* and other plants based on shared protein-coding genes with three priors. The mean divergence times of the nodes are shown next to the nodes, and the blue bars correspond to the 95% highest posterior density (HPD); *X*-axis unit, million years ago (MYA).

Phylogenetic analysis was carried out based on alignment of the concatenated nucleotide sequences of all the 20 chloroplast genomes through ML and BI (Figure 5). The topologies of the ML and BI trees were consistent, and most nodes had strong support values. Both trees indicated that *R. japonica* was sister to *F. multiflora* and formed an alone clade, which is consistent with traditional taxonomic classifications in *Flora of China*. In addition, the molecular data showed that the divergence time between *R. japonica* and *F. multiflora* was about 20.47 MYA (95% HPD = 13.82~34.46 MYA) (Figure 6). It could be inferred from the current data that *R. japonica* appeared earlier in South Korea than in China. At least before 2.22 MYA (95% HPD = 0.84~5.02 MYA), *R. japonica* had been delivered to China (Figure 6). The results also showed that all *R. japonica* clustered together with high resolution and were clearly divided into two major subclades, indicating that they may have relatively different homology and genetic relationships. Among them, one subclade is distributed in Zhejiang Province (*Rj. ZheJiangKJ*, *Rj. ZheJiangZYY*, and *Rj. ZheJiangU*), while the other is relatively scattered (Figure 5 and Figure 6).

### 3.6. Intraspecific Diversity and Genetic Structure of R. japonica

**Figure 7:**
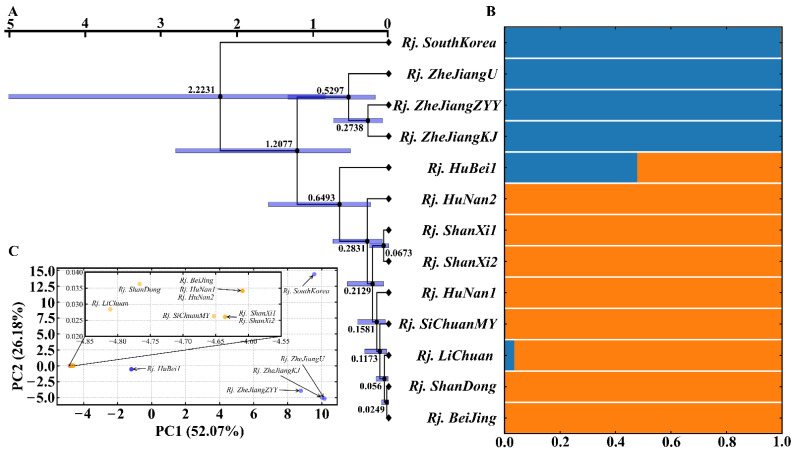
Intraspecific diversity and genetic structure of *R. japonica* based on whole chloroplast genomes: (**A**) phylogenetic tree; (**B**) population structure analysis with K = 2; (**C**) principal component analysis.

To further understand the possible group of *R. japonica* in China, molecular phylogeography was carried out. The samples were clearly divided into two clades according to phylogenetic relationships (Figure 7A), STRUCTURE analysis (Figure 7B), and PCA (Figure 7C). The results also showed that *R. japonica* in South Korea and Zhejiang Province in China had closer consanguinity. In addition, *Rj. HuBei1* processes 52.15% consanguinity, similar to that in South Korea and Zhejiang Province, and 47.85%, similar to other provinces and cities in China. During the propagation of *R. japonica* in China, the essential transition zone was Hubei Province, about 0.65 MYA (95% HPD = 0.24~1.59 MYA) (Figure 7A,B). This was followed by penetration into other regions of China, including Hunan, Shanxi, Sichuan, Beijing, Shandong, and so on. The two clades showed remarkable genetic differences and diverged at 1.21 MYA (95% HPD = 0.51~2.81 MYA) (Figure 7A). *R. japonica* appeared in several provinces in China within a similar period of time, reflecting its great adaptability and rapid invasion capacity.

## 4. Discussion

### 4.1. Comparison of Chloroplast Genomes in R. japonica

In this study, we sequenced and assembled three chloroplast genomes of *R. japonica* and performed a series of comprehensive comparative analyses with other published data. All the chloroplast genomes in *R. japonica* presented a typically quadripartite structure [22,23]. The chloroplast genome structure, codon bias, and gene order were conserved, consistent with buckwheat [20]. A slight variation was observed in chloroplast genome size and GC content (Table S2). However, some evident diversities in *R. japonica* were also detected. IR regions were considered the most conserved regions in the chloroplast genome [49], and its contraction or expansion was the main cause of chloroplast genome size variation [50]. In *Rj. ZheJiangZYY*, the whole chloroplast length was the shortest in the 13 accessions of *R. japonica*—it was only 163,183 bp (Figure 2). As 238 bp of *rps19* were located in the LSC region, it appeared that the IR region had contracted. In addition, the genes near the boundary of IR and LSC were *rpl2* and *trnH* rather than *rps19* and *trnH* in the other 12 *R. japonica*. We also found that the *accD* and *matK* genes were present as pseudogenes in *Rj. SiChuanMY*, which produce acetyl-CoA carboxylase subunit β and maturase K, respectively. The *accD* gene is also a pseudogene in some species [51,52] according to incomplete sequence and annotation.

### 4.2. Potential DNA Barcodes

Developing DNA barcodes based on differences in chloroplast genome sequences are widely used in species identification [20,53]. The comparison of whole chloroplast genomes by mVISTA indicated that the sequences of *R. japonica* were highly similar (Figure S1) but not as rich in variable sites as buckwheat [20]. The results showed that the chloroplast genomes of 13 *R. japonica* samples could be divided into two main categories. We further investigated the nucleotide diversities of these *R. japonica* (Figure 3). Based on the sequence variations, five protein-coding genes (*rps16*, *ycf3*, *rps12*, *psbN*, *ndhE*) and one nonprotein-coding gene (*trnG-UCC*) were selected, which were potentially useful for the population identification in *R. japonica* (Figure 3A,B). Moreover, six intergenic regions with high nucleotide diversity were found (Figure 3C). The intergenic regions of *trnS-GCU*/*trnG-UCC* and *ndhF*/*rpl32* were also identified in other angiosperms [16,53]. These results suggested that this DNA barcode could be used to identify *R. japonica* and conduct a phylogenetic tree. The results also indicated that mutation sites appeared clustered and mainly concentrated in the LSC region (Figure 3C). These polymorphic loci might be helpful for phylogenetic inference and population genetic studies of *R. japonica.*

### 4.3. Evolutionary Relationships

At present, a systematic study of genetic diversity and biogeographic evolution of *R. japonica* is still lacking. It is both desirable and helpful to investigate the systematical evolution and biogeographic relationships of this species. Although *R. japonica* was introduced to Europe, North America, and Australia with ornamental purposes in the 19th century and is now considered an invasive plant [54], it is native and widely distributed in China, Japan, and Korea [1]. Moreover, the medicinal use of *R. japonica* in China has a long history, first appearing in *Mingyi Bielu*, a famous medicinal book, 2000 years ago. In Japan, the *R. japonica* chloroplast genome region from the *rbcL* to the *accD* gene was obtained [55], and the intraspecific sequence variation in chloroplast regions reflected the species variety and geographical distribution. However, comparative analyses based on the whole chloroplast genome could provide a more comprehensive interpretation of intraspecific genetic structure and phylogenetic relationships than using only a few fragments [16].

The main aim of this study was to explore the characteristics of chloroplast genome divergence in *R. japonica*. The phylogenetic trees in our study showed the independent evolution of *R. japonica* species (Figure 5), which corroborates a separate taxonomic status for *R. japonica* in the Polygonaceae. The differentiation time of *R. japonica* and *V. vinifera* estimated based on the chloroplast genome was similar to that of the nuclear genome [56]. The results also revealed that *R. japonica* was divided into two subclades with significant genetic divergence (Figure 7B) and with Hubei Province as a transition zone. Located in the middle of the Yangtze River, an essential transport hub region in China, Hubei Province may serve as a crucial hub for *R. japonica* to further spread to other regions due to the frequent movement of personnel. The seeds of *Rj. LiChuan* were collected from Hubei Province and planted in our lab, and *Rj. LiChuan* and *Rj. HuBei1* had partial consanguinity from population genetic structure analysis (Figure 7B) with a relatively distant genetic distance (Figure 7A). This might be caused by the adaption of *R. japonica* or the different differentiation times in other regions of Hubei Province. In addition, the current data showed that *R. japonica* occurred earlier in South Korea than in China (Figure 7A), contrary to the notion that *R. japonica* originated in China [57]. Considering the wide availability of *R. japonica* across diverse regions of China, the debate on the origin of this medicinal plant is evidently not conclusive at this stage. From the tight timescale deduced from the chloroplast genomic data (Figure 7A), it is safe to say that this plant is indeed highly invasive [9].

## 5. Conclusions

*R. japonica*. is an essential medicinal plant. A large amount of genetic information was contained in the chloroplast genome. At present, systematic research into *R. japonica* evolution based on the chloroplast genome is still lacking. This study seems the first attempt to comprehensively investigate the chloroplast genome features and infer phylogeny for *R. japonica*. We assembled three complete chloroplast genomes, together with 10 other currently available data, to elucidate the evolutionary relationship of *R. japonica.* Our comparative analyses showed that chloroplast genomes of *R. japonica* are well conserved in terms of genome structure and codon usage, and there are two main subclades of *R. japonica* in China, with Hubei Province probably acting as the dispersal center region. The collected genomes and obtained genetic relationship in this study could contribute to further studies on population genetics, phylogeny, and conservation biology in Polygonaceae.

## Figures and Tables

**Table 1:** Chloroplast genome features of the three *R. japonica*.

	Length (bp)/GC Content (%)				Number of Genes (Unique)			
	LSC	IR	SSC	Total	Protein	tRNA	rRNA	Total
*Rj. BeiJing*	87,571	31,121	13,558	163,371 (37.5256%)	87 (79)	37 (30)	8 (4)	132 (113)
*Rj. LiChuan*	87,571	31,121	13,558	163,371 (37.5238%)	87 (79)	37 (30)	8 (4)	132 (113)
*Rj. ShanDong*	87,572	31,121	13,558	163,372 (37.5248%)	87 (79)	37 (30)	8 (4)	132 (113)

## Data Availability

The data used in our study have been submitted to NCBI GenBank (Accession Number: OP583946-OP583948).

## Supplementary Materials

The following supporting information can be downloaded at: https://www.mdpi.com/article/10.3390/genes13111979/s1, Figure S1: comparison of 13 *R. japonica* chloroplast genomes using mVISTA, with *Rj. BeiJing* as the reference. The *Y*-axis represents the percent identity within 50~100%. Gray arrows indicate the direction of gene transcription. Purple bars represent exons, blue bars represent untranslated regions (UTR), pink bars indicate conserved noncoding sequences (CNS), gray bars represent mRNA, and white peaks represent differences of genomics, Table S1: the sequencing depth of three *R. japonica*, Table S2: comparison of the complete chloroplast genomes for 13 *R. japonica*, Table S3: the RSCU values of 13 *R. japonica*, Table S4: the codon usage of 13 *R. japonica*.
