# Supplementary material for: A Comparative Phylogenetic Analysis on the Chloroplast Genome in Different Reynoutria japonica Populations
Source: Genes (Basel). 2022 Oct 30;13(11):1979. doi: 10.3390/genes13111979 (PMC9689882; doi:10.3390/genes13111979)
Supplement: Supplementary file 1 [file genes-13-01979-s001.zip › FigS1-mVISTA.pdf]

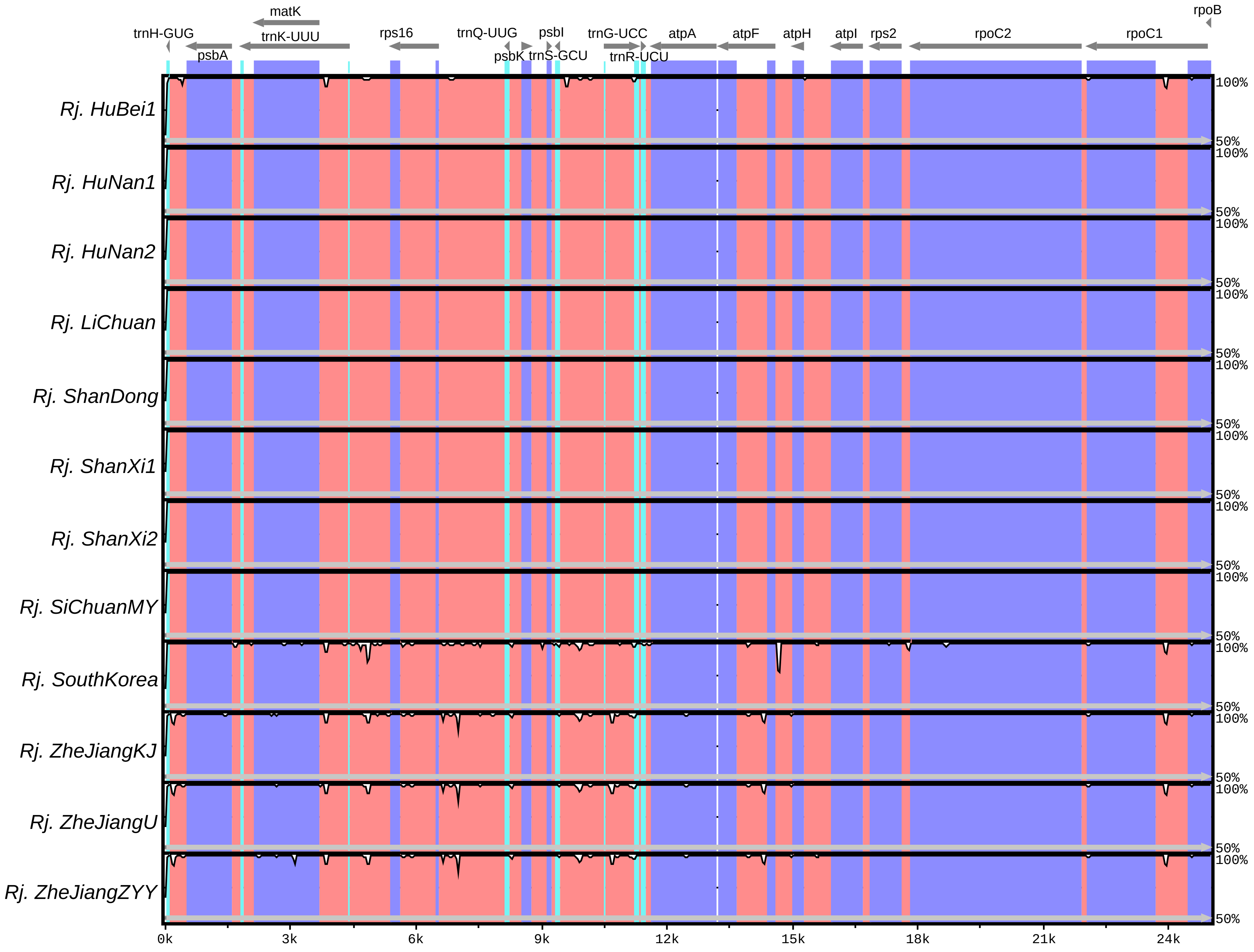

matK
trnH-GUG
psbA
trnK-UUU
rps16
trnQ-UUG
psbK
psbI
trnS-GCU
trnG-UCC
trnR-UCU
atpA
atpF
atpH
atpI
rps2
rpoC2
rpoC1
rpoB
Rj. HuBei1
Rj. HuNan1
Rj. HuNan2
Rj. LiChuan
Rj. ShanDong
Rj. ShanXi1
Rj. ShanXi2
Rj. SiChuanMY
Rj. SouthKorea
Rj. ZheJiangKJ
Rj. ZheJiangU
Rj. ZheJiangZYY
100%
50%
0k
3k
6k
9k
12k
15k
18k
21k
24k

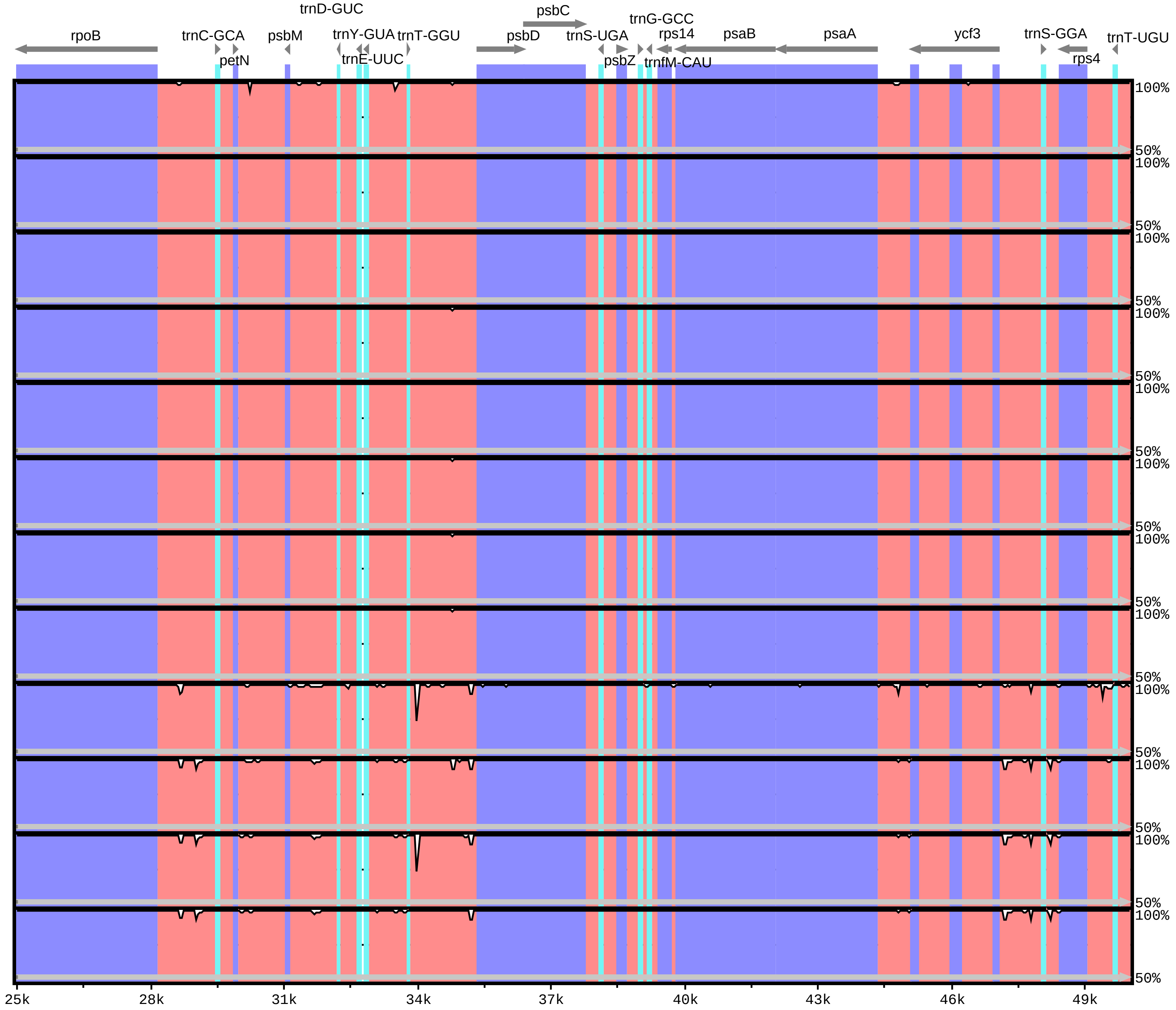

rpoB
trnC-GCA
petN
psbM
trnD-GUC
trnY-GUA
trnE-UUC
trnT-GGU
psbD
psbC
trnS-UGA
psbZ
trnG-GCC
trnfM-CAU
rps14
psaB
psaA
ycf3
trnS-GGA
rps4
trnT-UGU
100%
50%
25k
28k
31k
34k
37k
40k
43k
46k
49k

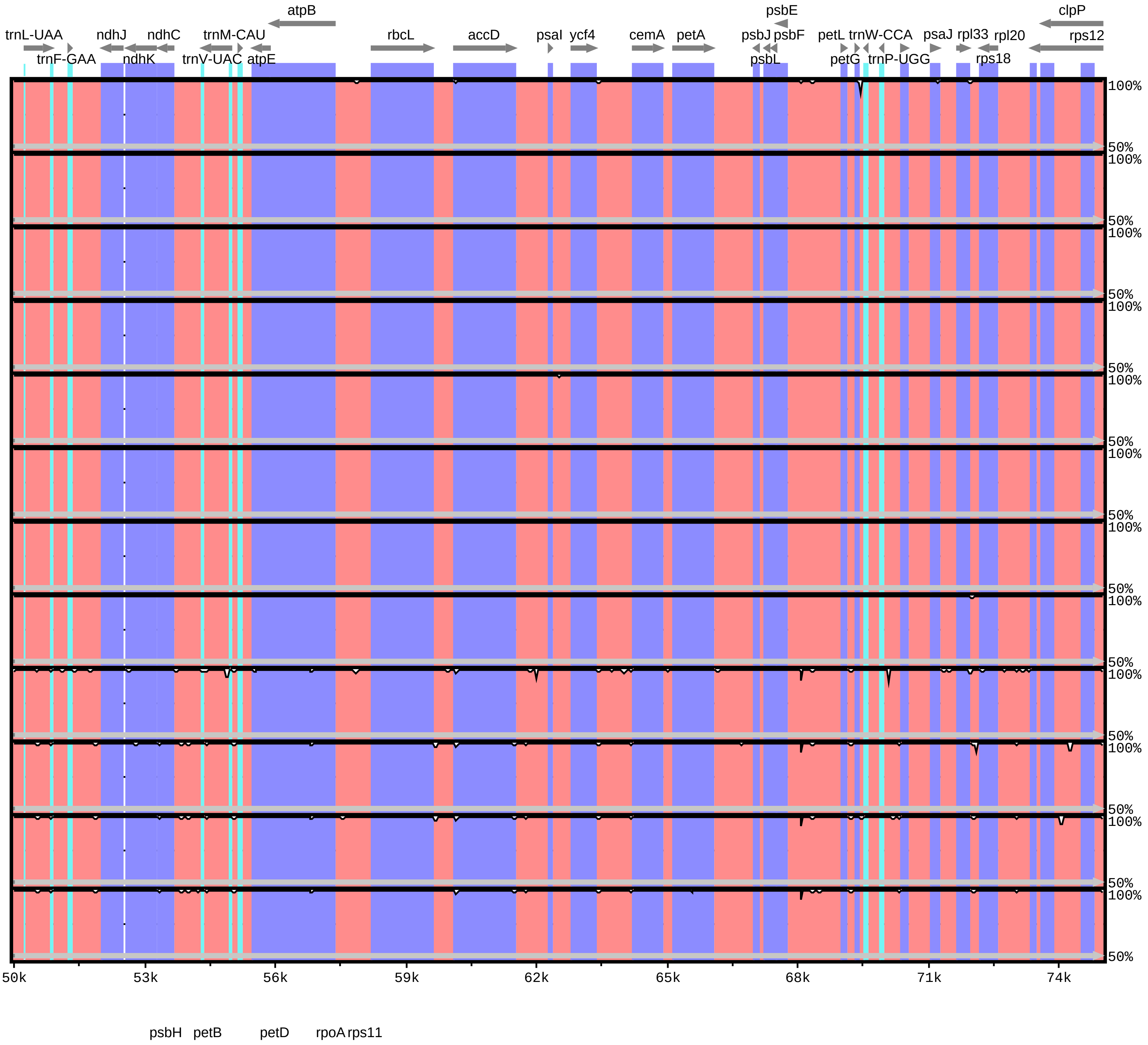

atpB
psbE
clpP
trnL-UAA
ndhJ
ndhC
trnM-CAU
rbcL
accD
psaI
ycf4
cemA
petA
psbJ
psbF
petL
trnW-CCA
psaJ
rpl33
rpl20
rps12
trnF-GAA
ndhK
trnV-UAC
atpE
psbL
petG
trnP-UGG
rps18
100%
50%
50k
53k
56k
59k
62k
65k
68k
71k
74k
psbH
petB
petD
rpoA
rps11

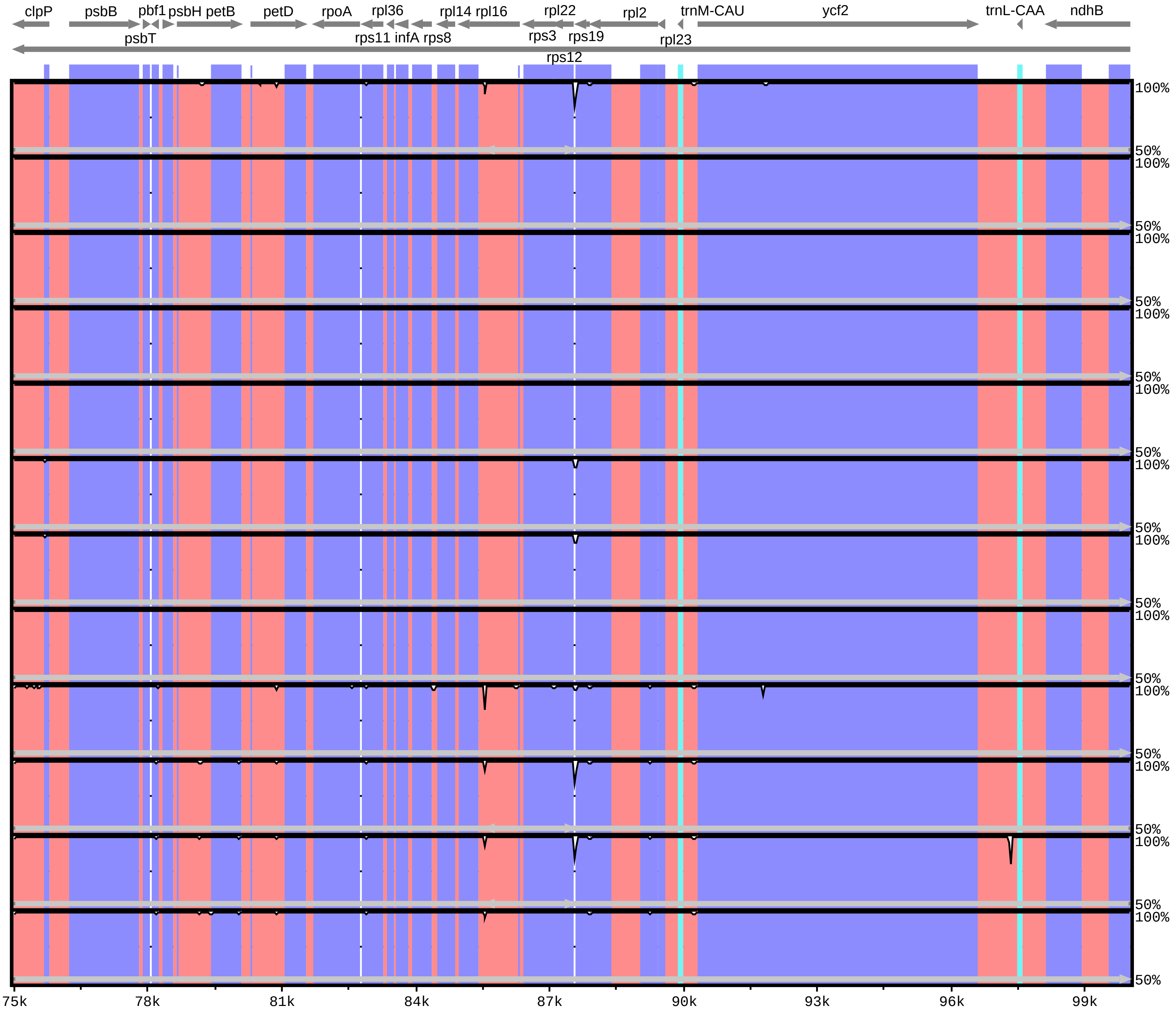

clpP
psbB
pbf1
psbH
petB
petD
rpoA
rpl36
rpl14
rpl16
rpl22
rpl2
trnM-CAU
ycf2
trnL-CAA
ndhB
psbT
rps11
infA
rps8
rps3
rps19
rpl23
rps12
100%
50%
75k
78k
81k
84k
87k
90k
93k
96k
99k

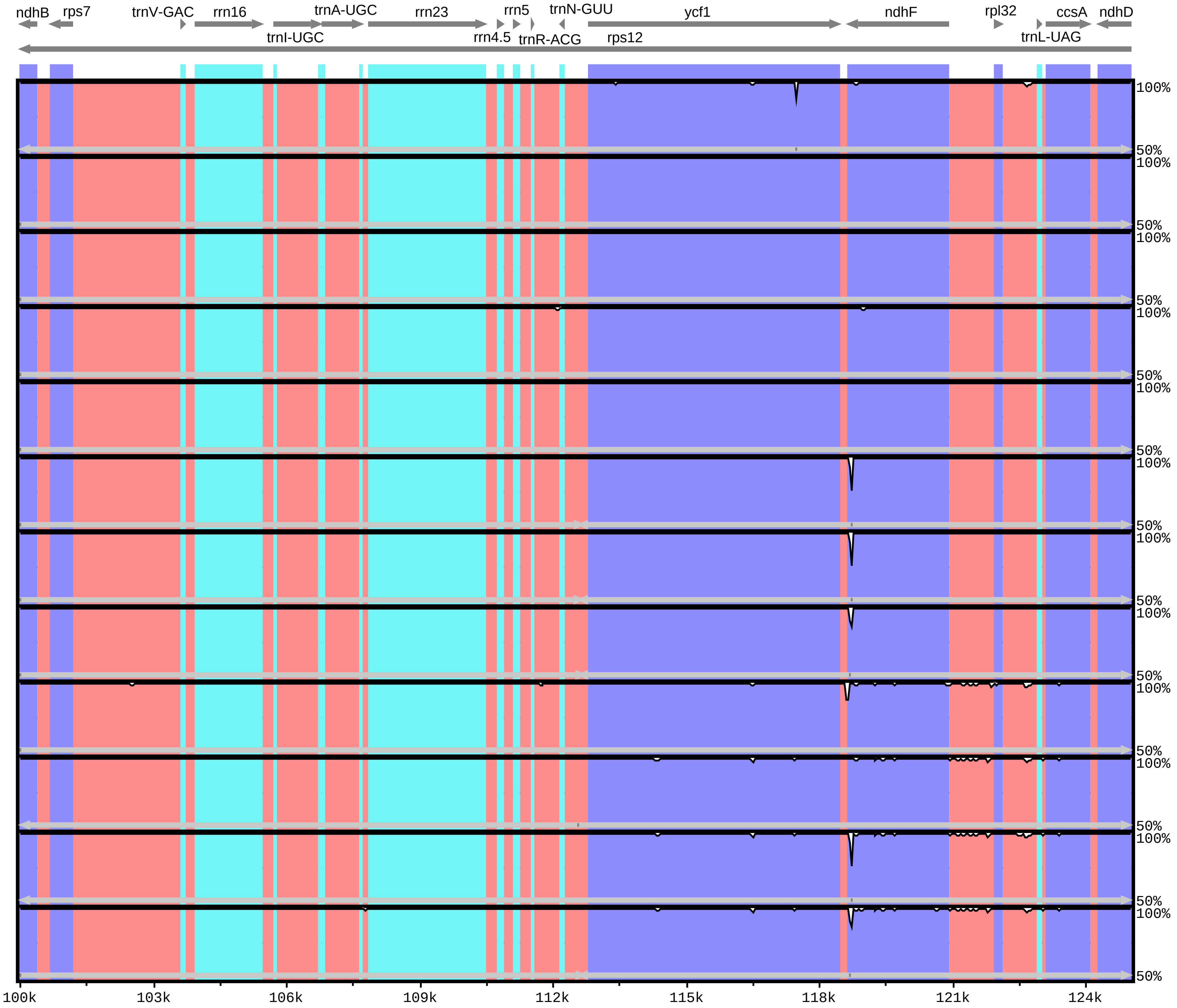
ndhB
rps7
trnV-GAC
rrn16
trnI-UGC
trnA-UGC
rrn23
rrn4.5
rrn5
trnR-ACG
trnN-GUU
rps12
ycf1
ndhF
rpl32
trnL-UAG
ccsA
ndhD
100%
50%
100k
103k
106k
109k
112k
115k
118k
121k
124k

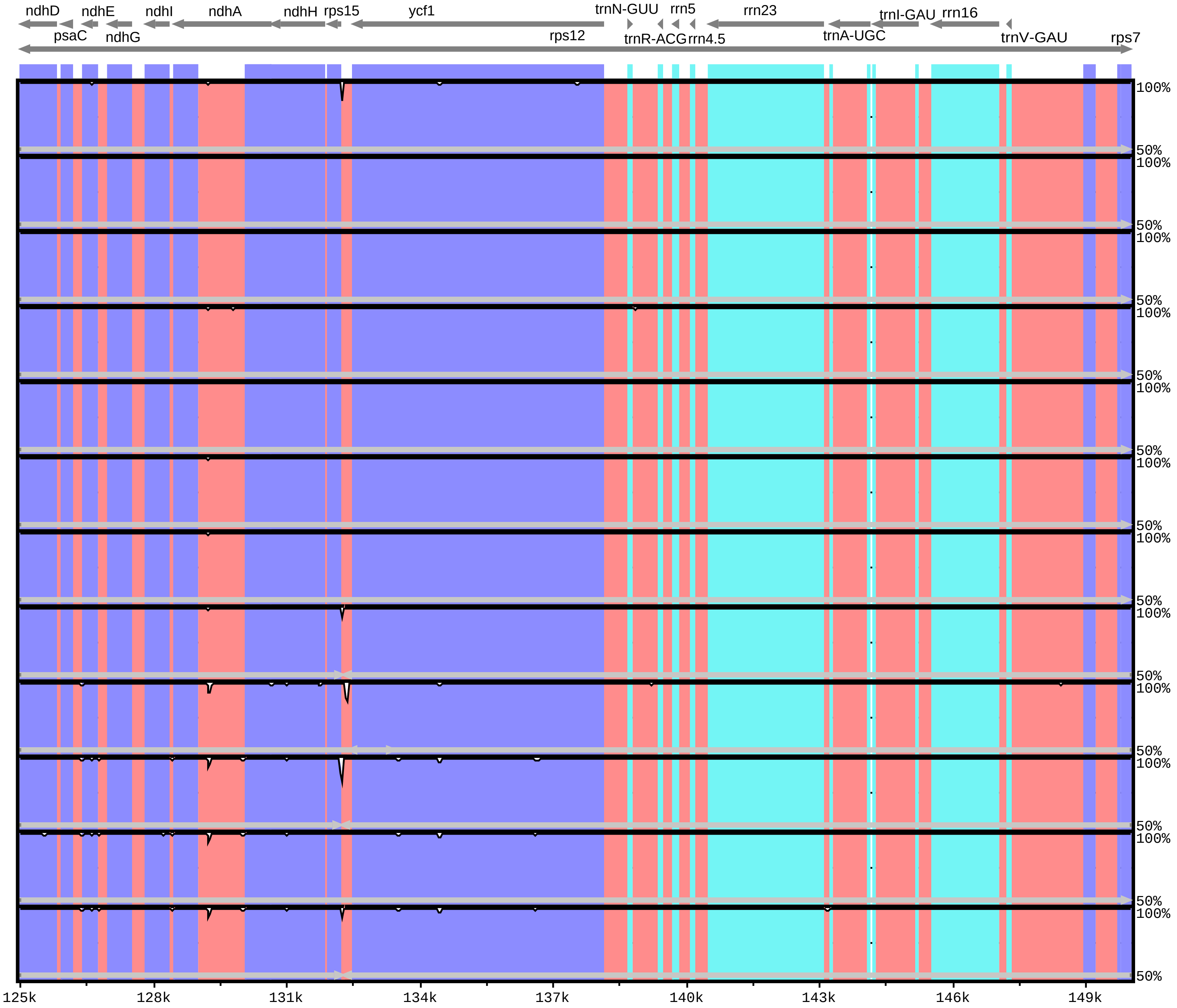
ndhD
ndhE
ndhI
ndhA
ndhH
rps15
ycf1
trnN-GUU
rrn5
rrn23
trnI-GAU
rrn16
psaC
ndhG
rps12
trnR-ACG
rrn4.5
trnA-UGC
trnV-GAU
rps7
100%
50%
125k
128k
131k
134k
137k
140k
143k
146k
149k

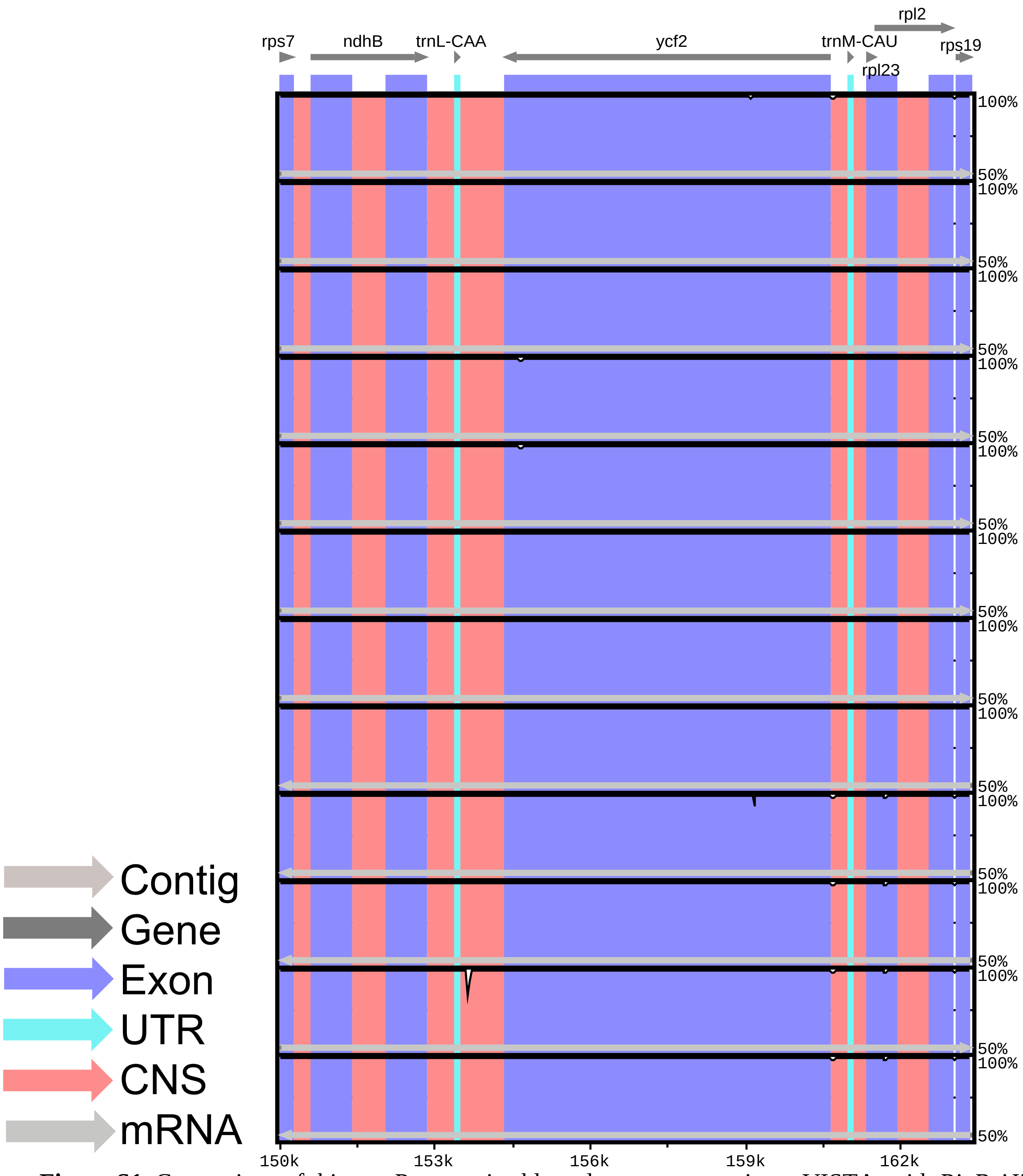

Figure S1. Comparison of thirteen *Reynoutria* chloroplast genomes using mVISTA, with *Rj. BeiJing* as the reference. The Y-axis represents the percent identity within 50~100%. Gray arrows indicate the direction of gene transcription. Purple bars represent exons, blue bars represent untranslated regions (UTR), pink bars indicate conserved noncoding sequences (CNS), gray bars represent mRNA, and white peaks represent differences of genomics.
